# Availability and distribution of safe abortion services in rural areas: a facility assessment study in Madhya Pradesh, India

**DOI:** 10.3402/gha.v8.26346

**Published:** 2015-03-20

**Authors:** Sarika Chaturvedi, Sayyed Ali, Bharat Randive, Yogesh Sabde, Vishal Diwan, Ayesha De Costa

**Affiliations:** 1 Department of Public Health and Environment, R D Gardi Medical College, Ujjain, India; 2 Department of Public Health Sciences, Karolinska Institutet, Stockholm, Sweden; 3 Department of Clinical Medicine and Public Health, Umea University, Umea, Sweden; 4 Department of Community Medicine, R D Gardi Medical College, Ujjain, India; 5 International Centre for Health Research, R D Gardi Medical College, Ujjain, India

**Keywords:** abortion, maternal health, India, JSY

## Abstract

**Background:**

Unsafe abortion contributes to a significant portion of maternal mortality in India. Access to safe abortion care is known to reduce maternal mortality. Availability and distribution of abortion care facilities can influence women's access to these services, especially in rural areas.

**Objectives:**

To assess the availability and distribution of abortion care at facilities providing childbirth care in three districts of Madhya Pradesh (MP) province of India.

**Design:**

Three socio demographically heterogeneous districts of MP were selected for this study. Facilities conducting at least 10 deliveries a month were surveyed to assess availability and provision of abortion services using UN signal functions for emergency obstetric care. Geographical Information System was used for visualisation of the distribution of facilities.

**Results:**

The three districts had 99 facilities that conducted >10 deliveries a month: 74 in public and 25 in private sector. Overall, 48% of facilities reported an ability to provide safe surgical abortion service. Of public centres, 32% reported the ability compared to 100% among private centres while 18% of public centres and 77% of private centres had performed an abortion in the last 3 months. The availability of abortion services was higher at higher facility levels with better equipped and skilled personnel availability, in urban areas and in private sector facilities.

**Conclusions:**

Findings showed that availability of safe abortion care is limited especially in rural areas. More emphasis on providing safe abortion services, particularly at primary care level, is important to more significantly dent maternal mortality in India.

The recent global burden of disease estimates (1) on the causes of maternal deaths reported that 15% (43,000 of 293,000) of global maternal deaths were attributable to unsafe abortion. However, the authors cautioned that this number is likely to be an underestimate given the selective underreporting of abortion-related deaths. Recent estimates indicate that 44 million abortions occurred globally in 2008, 86% of which occurred in developing countries, and half of which were unsafe (2). Estimates of the number of abortions in India vary widely. Data from government statistics on family welfare in India record that 620,472 abortions took place in 2010–2011 at approved institutions (3). The Consortium on National Consensus for Medical Abortion in India argues that these numbers are gross underestimates because hospitals record only legal and reported abortions (4). The consortium estimates 11 million abortions in the country every year, and 20,000 deaths from unsafe abortions (5). Only 40% of abortions in India are considered safe (6).

Although the uptake of contraceptive services has been steadily increasing, national surveys indicate that it is still low; only 55% of currently married women use a contraceptive to delay or avoid pregnancy (7). Several studies from India report that the desire to limit family size and to space the next birth are the main reasons for seeking abortion, as mentioned by a majority of abortion seekers (8, 9), indicating a clear link between poor access to temporary contraception and abortion.

Despite the early legalisation of abortion in India in the early of 1970s, safe and legal abortion remains largely unavailable. A task force commissioned by the Indian Council of Medical Research found more than twice as many illegal abortions occurred as legal ones (10). Another estimate in 1991 contends a rate of three illegal abortions to one legal abortion in rural areas and a corresponding ratio of 4–5:1 in urban areas (11). A third subsequent estimate puts the figure at eight illegal abortions for every legal one (12). In India, the government had made significant investments in the public health system through the National Rural Health Mission (NRHM) (13) by way of improvements in infrastructure and increased recruitment of doctors and nurses. These investments and programs aim to also reduce maternal deaths while strengthening the health system, and they provide an opportunity to increase the availability of functional abortion services in the public sector. This is particularly relevant given that abortion ranks among the five top causes of maternal mortality in India (14). Abortion services have remained predominantly in the heterogeneous private healthcare sector (15). In the present paper, we studied the availability of safe abortion care at public facilities (5 years after the NRHM) and private facilities in three districts of a large Indian state, Madhya Pradesh (MP). We also studied the differences between public and private facilities that provide these services. Further, we map the locations of these facilities to study geographic access particularly for rural women.

The study is important because it provides information on the existing access to safe abortion (which is still an important cause of maternal death) under the NRHM and in the private sector. This information is important while considering strengthening access to services or forming public private partnerships to enhance access to services, given that unsafe abortion is a preventable cause of maternal mortality and contributes to 8% of India's current maternal deaths.

## Methods

### Settings

The study was conducted in the large, central Indian state of (MP); over two thirds of MP's 72 million population is rural (16). A third of all inhabitants live below the poverty line (17). Infant mortality stands at 59 per 1,000 births, which is the highest in India. Point estimate for MMR currently stands at 277 maternal deaths per 100,000 births (18). The public sector is the dominant provider of obstetric services in the province. The private health sector is small, concentrated in urban areas. In MP, 10% of all institutional deliveries occur in the private sector (19). The public health system has a three-tiered network of facilities – each district in the province has an apex district hospital (DH) which is intended to be a tertiary level hospital handling cases arriving directly or referred from community health centres (CHCs) that are meant to be secondary care facilities within districts. CHCs in turn receive cases arriving directly or referred from primary health centres (PHCs) in the periphery. Safe abortion services are included in the package of Basic Emergency Obstetric Care (BEmOC) services deemed to be available at all three levels of facilities. In MP, as in the rest of the country, the law mandates the state to provide abortion services at all public hospitals. The Annual Health Survey for Madhya Pradesh reported 45% of abortions occurred at health facilities (18), indicating a potentially large utilisation of unsafe abortion services. In MP, there are no stand-alone abortion care centres; abortion care is available in maternity centres and medical abortion is not in routine practice in public facilities.

### Study districts and facilities

Districts are administrative units within a province. Each district has a population between 1 and 1.5 million. Of the 51 districts in MP, three heterogeneous districts were selected for this study based on their geographic location and differing socio-economic level of development [as indicated by human development indices (HDI)]. One of the three districts was a tribal district with an HDI of 0.4 and an urban population of 29%; one was a less developed district with an HDI of 0.5 and 22% urban population, while one was a better developed district with an HDI of 0.6 and 42% urban population. Study facilities were public or private facilities that reported at least 10 deliveries a month in the last year. The list of facilities providing delivery care in the district was obtained from the district authorities. Snowballing was also used to complete this list by enquiring for names of delivery facilities in the vicinity of the listed facilities.

### Data collection

Medically trained researchers surveyed each study facility as part of a larger project between February 2012 and April 2013. The team visited each study facility without prior notice of the visit. The chief of the facility was interviewed to know if the facility had the ability to provide abortion care and whether the facility had provided abortion service at least once in the recent 3 months. Service statistics regarding abortion care (surgical) were obtained from records of each facility for the last 6 months.

#### Mapping

The geo-referenced data of the study districts was entered in ArcMap version 10. For geo-referencing, Survey of India topological sheets with a 1:50,000 scale were used. Readings of hand held global positioning system (GPS) at random locations were used to cross verify geo-referencing. The geo-referenced data included 1) the digital boundary maps of the study districts, which were retrieved from the office of Survey of India; 2) locations of all included study facilities.

Ethical approval to conduct the study was obtained from the Institutional Review Board at R D Gardi Medical College, Ujjain, India.

### Analysis

**Fig. 1:**
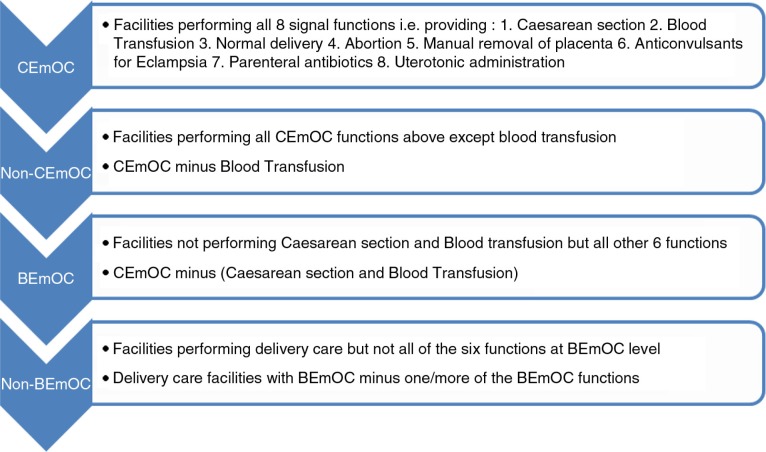
Criteria for classification of delivery care facilities in this study.

The performance of Emergency Obstetric Care (EmOC) signal functions at the facilities was ascertained to allow their classification into BEmOC or Comprehensive Emergency Obstetric Care (CEmOC) facilities based on WHO classification (20, 21). CEmOCs were facilities that performed all eight signal functions including caesarean section and blood transfusion. We included a category ‘Non-CEmOCs’ which consisted of facilities that provided caesarean section services (but not blood transfusion) but failed to provide all eight CEmOC signal functions. Non-BEmOCs were the facilities that failed to provide six basic EmOC signal functions (Fig. 1).

As with the assessment of level of EmOC functioning, performance of an abortion at least once in the last 3 months was used as an indicator of functional abortion services. The geographic distribution of abortion care facilities was visualised using ArcMap version 10.

## Results

### Characteristics of facilities and abortion service capacity

The three districts had 99 facilities that conducted >10 deliveries a month: 74 in public, 25 in private sector (of which three private facilities refused to participate).

Altogether five of the study facilities qualified as CEmOC facilities: one in public and four in private sector. While all the private facilities performed caesarean section (CS), most of the public facilities were at the less than BEmOC level, that is, conducted deliveries but did not perform all six basic signal functions (Table 1).

**Table 1:** Distribution of the 96 obstetric care facilities studied

Facilities	Public *N*=74	Private *N*=22	Total *N*=96
CEmOC	1	4	5
Non-CEmOC (CS)	4	18	22
Less than BEmOC	69	0	69
Facilities reporting ability to perform surgical abortion	24	22	46
Facilities where a surgical abortion was performed during the last 3 months			30
CEmOC	1	4	5
Non-CEmOC	4	13	17
Non-BEmOC	8	0	8
Number of surgical abortions performed in the last 6 months	364^a^	314	678^a^

a Data could not be retrieved for one large public hospital performing surgical abortions routinely.

Overall, 48% (*n*=46) of facilities reported an ability to provide safe abortion services. Of public centres, 24 (i.e. 32%) reported the ability compared to 100% among private centres. However, the proportion of facilities where surgical abortion was actually performed in the last 3 months was 18% for public facilities and 77% for private facilities.

Of the 30 facilities that had performed a surgical abortion in the last 3 months, five (one public and four private) were CEmOC facilities, 17 were non-CEmOC (four public and 13 private). Only eight public facilities, which functioned at less than BEmOC level, had performed abortions in the last 3 months.

All DHs, 47% of secondary level facilities and only 3% of primary level facilities had provided one abortion service in the last 3 months.

### Differences in functionality between facilities

Facilities that performed abortions were significantly more likely to have better infrastructure and staff availability. As seen in Table 2, facilities that performed abortions were better equipped in terms of availability of operation theatre (86% vs. 12%), equipment for dilatation and curettage (93% vs. 37%), manual vacuum aspiration sets (90% vs. 17%), and also availability of obstetricians (76% vs. 10%).

**Table 2:** Characteristics of facilities by performance of abortion in the last 3 months and by ownership types of facilities

	Facilities that performed surgical abortion in the last 3 months *N*=30			Facilities that did not perform surgical abortion in the last 3 months *N*=66		
	
Facility	Public *N*=13	Private *N*=17	Total *N*=30 (%)	Public *N*=61 (%)	Private *N*=5 (%)	Total *N*=66 (%)
Labour room	13	17	30 (100)	60	5	65 (99)*
Operation theatre	9	17	26 (86)	3	5	8 (12)*
Obstetrician	6	17	23 (76)	2	5	7 (10)*
Non-obstetrician doctor^a^	7	0	7 (24)	41	0	41 (62)
Functional manual vacuum aspiration equipment	10	17	27 (90)	8	3	11 (17)*
Functional dilatation and curettage equipment	11	17	28 (93)	19	5	24 (37)*

a Facilities with a non-specialist doctor if there was no obstetrician

* *p*<0.05.

The differences in key infrastructural parameters between public and private institutions that performed abortions in the last 3 months are also shown in Table 2. All private facilities performing abortions had an obstetrician, while few such public facilities had non-obstetrician doctors and did not have an operation theatre.

### Distribution of abortion care facilities

**Fig. 2:**
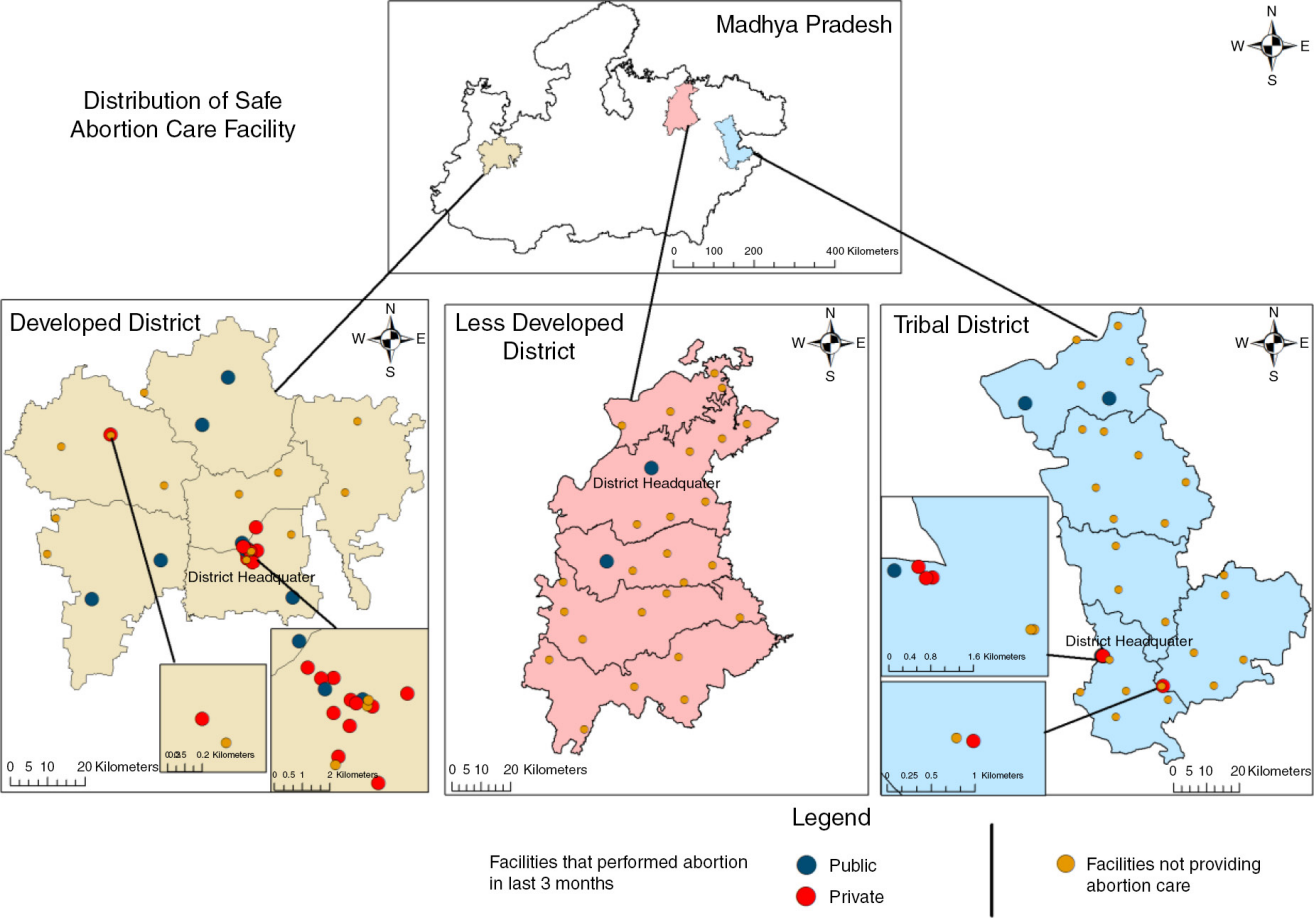
Distribution of abortion facilities in three districts of Madhya Pradesh.

The GIS maps (Fig. 2) show a concentration of abortion care facilities near district head quarters which are urban areas. The figure also shows that some public facilities providing abortion care are available in rural areas, while of the few private facilities with these services, hardly any (three out of 22) are in rural areas. The differences in the availability of abortion facilities among districts are evident from the maps – the better developed district had better availability of these facilities than the less developed and tribal districts. The private sector has rare presence in rural areas and is non-existent in the less developed district as regards abortion care facilities.

## Discussion

Our findings indicate that in terms of number of facilities, the numbers of private and public facilities providing abortion services in the study districts are similar. Although service statistics showed a similar number of abortions were performed in public and private facilities, the number for public facilities is likely to be much higher considering the unavailability of data at a large public hospital where surgical abortion is routinely provided. Reports based on nationwide surveys indicate that the private sector is the dominant provider of abortion services (21). The latest available national district level survey (7) indicates the 54% of all induced abortions were in the private sector, 38% in the public sector and the remainder at home. However, the definition of private sector used in these surveys is wider than the one followed in the study, which focuses on facilities that provide intrapartum care to at least 10 parturients a month. The proportion of private sector abortions we found in this study is somewhat lower at 46% and could be because of the type of facilities included or because of the sparse availability of private sector facilities in two of the three study districts.

An important element that distinguishes facilities that provide abortion services, regardless of sector, is the level of functionality. Private facilities on the whole had higher levels of functionality; all 22 in our study had the ability to perform caesarean sections (four were CEmOC, 18 were Non-CEmOC facilities) whereas only 7% (*n*=5) of public sector facilities did (69 performing below BEmOC levels). These five public facilities also provided abortion services. Only a very small proportion of public facilities with lower levels of functionality provided any abortion services; of all primary centres, only 3% provided these services. This is similar to findings documented in 2003 by the national facility survey (22) when the proportion of PHCs capable of performing abortions was 6.1% for India as a whole and 2.7% for MP. Therefore, the problem of less availability of safe abortion services for rural women still remains in 2014.

Despite the number of facilities being similar, studying the geographic locations of facilities providing abortion services, clearly demonstrates that the public sector importantly provides coverage of abortion services in areas where the private sector does not have a presence. Our study shows a clustering of private facilities in the socio-economically better-off district (relative to the other two districts), and concentrated in urban areas. The two poorer districts lack any private abortion services. Urban areas are well served by public and private abortion facilities; however, the interior areas of the districts rely almost exclusively on public sector abortion services.

There is a strong need to strengthen the public provision of abortion services, particularly in these areas to improve access for rural populations. At the same time, the opportunity for such strengthening also exists in the context of the reforms through the NRHM which also aim to reduce maternal mortality. The government has recently indicated its commitment to the provision of abortion services at primary care level by announcing that 25,000 PHCs will be supported to provide such services (23). The level of functionality of these facilities needs attention and improvement. This includes among other efforts, the training of staff competent in providing services at the primary healthcare facility level and the provision of necessary infrastructure and equipment. While such strengthening is essential, and will probably occur over the medium term, given that a number of reports suggest that 75% (or more) of women seeking abortion services are in their first trimester (24–26) , other strategies can be deployed to make available abortion to rural women at primary healthcare level. These include the provision of medical abortion services at primary healthcare level. A recent large cohort project on medical abortion in Jharkhand and Bihar provinces in India (27) indicated a high degree of successful abortions (95%) even when administered by physicians trained in non-allopathic (ayurvedic) systems of medicine. A recent study from South India (28) has reported preference of rural women to access medical abortion from primary healthcare centres in the public sector and accept the service from mid-level providers like nurses. While this preference may not be representative of those made by rural women from states with weaker health systems, they are nevertheless indicative of a willingness to access medical abortion from a functional primary healthcare centre. Manual vacuum aspiration has also been used successfully for first trimester abortion at primary healthcare level (26) even when performed by nurses in India (29). The procedure has been shown to be safe, cost effective, and can be performed easily in basic facilities. It has been advocated by the WHO as a preferred method for uterine evacuation under 12 weeks gestation (30). The NRHM has recently brought out guidelines for abortion care (31) that refer to these two procedures. However, ensuring the availability and performance of medical abortion and vacuum aspiration, especially at BEmOC level facilities in the periphery, will provide access to safe abortion for thousands of rural women. Barriers to the implementation of these services at primary care level need to be addressed. It is important to consider that providing abortion care at facilities women routinely access for health needs, especially childbirth-related care, can ease access to abortion services and improve their utilisation. Hence, we strongly recommend delivery facilities be strengthened to also provide abortion care. Abortion is still one the five major causes of maternal deaths in India, and the government needs to make more of an impact on the provision of safe abortion services. The potential for increasing availability of safe abortion services in rural areas through provision by trained nurses needs to be explored. It is a step forward that the current amendments to the Medical Termination of Pregnancy Act in India are considering allowing nurses to provide abortion care.

The scope for public–private partnerships for the provision of safe abortion in the setting merits careful consideration because of the geographic location of the private sector in these districts. While the location and type of private sector is different in different parts of the country, in the study province the qualified private sector is located in large urban centres, where public sector options also exist. A partnership with urban private providers might help increase choice for urban and periurban residents. While it will not reduce geographical access barriers for rural women, partnership with the private sector is still an important consideration for abortion, as previous studies have reported that women prefer to use private facilities because of privacy (28). Financial barriers to access could also be addressed in such a partnership. Partnerships could be explored as an option in districts where there is a presence of private qualified safe abortion providers; however, caution is suggested given past reports of limited benefits of such partnerships to poor rural women. There has been the recent establishment of a public–private partnership program in Bihar, Yuktiyojana (32), to increase access to safe abortion services. Private facilities are required to have either a qualified gynaecologist or a doctor trained to provide abortion, as well as a functioning labour room and operating theatre. There were 49 such private facilities accredited under the Yukti program across Bihar (33); although the location of these facilities is not reported, it is very likely that most are in urban centres. Over an 18-month period 10,700 women were provided abortion care, two thirds of whom were rural (33). However, in large areas of our study districts in MP (even in one whole district), there was no private facility providing abortion services; therefore, the public sector is the major provider. Hence a focus on strengthening primary care centres to provide abortion is critical in poorer/remote districts where options to accessing such care in private facilities are sparse or absent.

### Limitations

This study did not focus on medical abortion in the studied facilities, and so is unable to comment on the availability of this alternative at the different facilities. Abortion numbers from facilities are based on reported service statistics over a 6-month period and these numbers could vary year on year. Also given the inclusion criteria of our study facilities, facilities that are principally abortion providers but do not provide intrapartum care would have been excluded. However, we do not expect there to have been any such facilities. Also the informal private sector, where a large number of abortions occur, is not considered in this paper. Generalizability of our results to other districts may be limited as the distribution of the private sector is different across districts; also the level of public sector abortion service provision is likely to vary across districts. While interpreting our results, we report low availability of abortion care services based on the government norm that these services ought to be available, as part of BEmOC, at all facilities providing delivery care. However, it is important to note that our results are based on facility survey and not on population level data on access to abortion services.

## Conclusions

In conclusion, while both the public and private sectors provide numerically similar abortion services in the study districts, in terms of numbers of facilities and of abortions performed, it is clear that the public sector is the sole provider of such services outside large urban centres. The level of provision in PHCs continues to be low, with serious implications to access for rural women. While strengthening of service provision in terms of human resources and infrastructure is important, other strategies like the provision of medical abortion through mid-level providers and active promotion of manual vacuum aspiration at PHC are key to improving access. Public–private partnerships are options that can be cautiously exercised only in selected areas where there is some level of private provision of safe abortion services. Unsafe abortion being among the five major causes of maternal deaths in India, the national health mission needs to place more emphasis on strengthening facilities to provide safe abortion services, particularly at primary care level, to more significantly dent maternal mortality.
